# Automatic Classification System for Periapical Lesions in Cone-Beam Computed Tomography

**DOI:** 10.3390/s22176481

**Published:** 2022-08-28

**Authors:** Maria Alice Andrade Calazans, Felipe Alberto B. S. Ferreira, Maria de Lourdes Melo Guedes Alcoforado, Andrezza dos Santos, Andréa dos Anjos Pontual, Francisco Madeiro

**Affiliations:** 1 Escola Politécnica de Pernambuco, Universidade de Pernambuco (UPE), Recife 50720-001, Brazil; 2 Unidade Acadêmica do Cabo de Santo Agostinho, Universidade Federal Rural de Pernambuco (UFRPE), Cabo de Santo Agostinho 54518-430, Brazil; 3 Departamento de Clínica e Odontologia Preventiva, Universidade Federal de Pernambuco (UFPE), Recife 50670-420, Brazil

**Keywords:** automatic classification system, endodontic lesion, deep learning, Siamese concatenated network

## Abstract

Imaging examinations are of remarkable importance for diagnostic support in Dentistry. Imaging techniques allow analysis of dental and maxillofacial tissues (e.g., bone, dentine, and enamel) that are inaccessible through clinical examination, which aids in the diagnosis of diseases as well as treatment planning. The analysis of imaging exams is not trivial; so, it is usually performed by oral and maxillofacial radiologists. The increasing demand for imaging examinations motivates the development of an automatic classification system for diagnostic support, as proposed in this paper, in which we aim to classify teeth as healthy or with endodontic lesion. The classification system was developed based on a Siamese Network combined with the use of convolutional neural networks with transfer learning for VGG-16 and DenseNet-121 networks. For this purpose, a database with 1000 sagittal and coronal sections of cone-beam CT scans was used. The results in terms of accuracy, recall, precision, specificity, and F1-score show that the proposed system has a satisfactory classification performance. The innovative automatic classification system led to an accuracy of about 70%. The work is pioneer since, to the authors knowledge, no other previous work has used a Siamese Network for the purpose of classifying teeth as healthy or with endodontic lesion, based on cone-beam computed tomography images.

## 1. Introduction

In Dentistry, imaging exams perform an important role in diagnosis support, because they help the dentist to obtain important information about dental tissues and facial bones, such as roots, which are anatomical regions inaccessible by means of the usual clinical examination [1].

Among the imaging techniques widely used in Dentistry, one can mention cone-beam computed tomography (CBCT), which refers to a diagnostic imaging method that portrays structures from three dimensions [2,3]. The CBCT performs a volumetric analysis of the region of interest; for this reason, it is possible to obtain a more faithful representation of the patient’s dental arch. The cone-beam computed tomography is considered a highly precise imaging exam [4,5]. The most frequent medical reasons for taking a dental tomography are for the suspicion of cysts and tumors; analysis of the roots’ proximity of the injured teeth and the mandibular canal or the inferior alveolar nerve; and for investigating periapical lesions, either for detecting the lesion’s location or for previous treatments’ adequacy verification, in which an endodontic evaluation is carried out [6,7].

The growing demand for imaging exams is notorious, which is supported by their usefulness in the detection of abnormalities and in treatment planning [8]. Nevertheless, the analysis of such images is not trivial; for this reason, it is often performed by experienced radiology specialists to obtain an adequate diagnosis [9,10]. To overcome this challenge, computer systems have been proposed as a support tool for analyzing image exams, with the advantage of performing fast, accurate, and objective tasks such as segmentation, detection, and classification [11,12].

Deep learning has shown to be a viable field of study in the development of expert systems for the aforementioned tasks related to images, especially the so-called convolutional neural networks (CNN), as they perform well in image pattern recognition [13,14]. Therefore, artificial intelligence techniques are used in health sciences as an alternative to aid image-based diagnosis. In many cases, it is possible to reduce the time needed to perform the diagnosis and even increase the accuracy when compared to the evaluation performed by specialists [15].

In dental practice, there is a consensus about the difficulty in analyzing imaging exams; however, as the human tooth is essentially composed by the crown—a clinically visible region, and by the root—a structure that can only be evaluated by imaging methods, it becomes indispensable in the dental routine to request imaging exams for a complete anamnesis of the patient [16]. Furthermore, the dental arch is divided between mandible and maxilla, which indicate the lower and upper region, respectively; according to specialists, the visual analysis in the maxilla region is even more complex [17,18]. Periapical lesions correspond to an inflammatory response that manifests itself in the apex of the tooth after necrosis of the pulp tissue, and which occurs frequently, but can be difficult to detect, especially when the lesions are small and located in the maxilla [19]. In general, to reach the diagnosis, specialists request CBCT and evaluate tomographic sections, because, depending on the lesion, it can be more visible on one section compared with another; so, analyzing more than one section makes the evaluation more precise [20,21]. Further, specialists may use more than one plane to enhance the anamnesis’ accuracy. Concerning periapical lesion detection, it is usual to analyze the sagittal and coronal planes [22]. As far as we are aware, there are no previous published works in literature that explore both planes to classify the presence or absence of periapical lesions in CBCT images. In fact, few papers focus on periapical lesions while most of them are for caries or periodontal bone loss classification. Therefore, there is a lack of works addressing this issue. Additionally, if we consider the ones that address the issue, we observe that they use a small dataset [23,24] or they use only one plane [25], which suggests that there is room for obtainment of better results.

This paper introduces a new automatic classification system for the dental diagnostic in cone-beam computed tomography, in which the coronal and sagittal slices are considered for the detection of periapical lesions. In order to use pairs of images (i.e., both coronal and sagittal slices of a single tooth) as inputs for the machine learning model, a system that uses a Siamese Network is proposed [26]. The present work uses the Siamese Network not to compare two images, but to extract characteristics from both at the same time. It is important to highlight that this approach is innovative in the sense that, according to the authors’ knowledge, no other published paper in Dentistry uses more than one plane in the deep learning model. The proposed framework uses two planes for the classification task (presence or absence of periapical lesion). Transfer learning techniques with the DenseNet-121 [27] and VGG-16 [28] networks are also used to develop the classification system. To the authors’ knowledge, no other study has been found in the literature that uses deep learning and pairs of dental images for endodontic diagnostics.

### Related Works

Pattern recognition in dental images has shown a growing development due to its ability to assist the analysis of exams [29]. Therefore, there has been an increase in scientific production on this particular subject in recent years. One may highlight the following related areas: forensics, in the identification of bodies through the dental arch in serious accidents [30]; Forensic Dentistry, to estimate the age of individuals and verify the age of criminal majority [31]; Implant Dentistry, for the detection of teeth and implants [32]; and diagnosis, in which the objective is to provide a classification of teeth—for instance, in two classes, with or without pathology [33]. In the next paragraphs, related works are presented regarding machine learning techniques applied to dental-aided diagnosis.

In 2018, Choi, Eun, and Kim [34] presented a system for automatic detection of proximal caries, which are considered difficult to diagnose due to the low quality of the images. To do so, they used periapical radiographs and convolutional neural networks in a proposal comprising four steps: horizontal alignment, probability map generation, crown extraction, and refinement. In 2020, Haghanifar, Majdabadi, and Ko [35] developed an automatic classification system based on deep learning and transfer learning techniques to detect carious lesions with a database of 470 panoramic radiographs. In 2021, Leo and Reddy [36] proposed a hybrid system with an artificial neural network and a deep network to detect the presence of caries and the extent of contaminated tissue, from the stages of preprocessing; segmentation; extraction of attributes; and, finally, classification.

In the work of Kim et al. [37], the convolutional neural network DeNTNet is used to detect periodontal bone losses in 12,179 panoramic radiographs, which occur as a consequence of periodontitis, a serious disease that corresponds to an inflammation of the periodontal bone, and, if not detected early, can lead the patient to tooth loss. In the paper, a method was presented to identify the presence or absence of periodontal lesion, as well as the numbering of the affected tooth. In 2018, Lee et al. [38] presented a system for the diagnosis and prediction of periodontally compromised teeth, which was implemented by combining a pretrained deep CNN and a self-trained network, using periapical radiograph images.

Regarding the detection of periapical lesions, Setzer et al. [23] presented a study based on deep learning, with the U-Net architecture, to perform an automated segmentation of CBCT scans of 20 patients in order to detect endodontic lesions. With the same objective of the aforementioned article, Zheng et al. [24] proposed a new approach to the U-Net Network, which is the Dense U-Net, in which CBCT scans from 20 patients were used. Endres et al. [25] conducted a study to evaluate the performance of a convolutional neural network, based on image segmentation using U-Net architecture, to detect periapical radiolucencies. They used 2902 panoramic radiographs that were evaluated by 24 dental and maxillofacial surgeons. The CNN method was compared to human evaluation and presented superior performance over 14 out of the 24 experts. Ezhov et al. [39] published a work describing an experimental study that uses a deep-learning-based system to aid dentists to detect periapical lesions. The authors compare recall and specificity results between aided and unaided groups of dentists while performing a clinical evaluation. It is important to mention that the authors do not present the results obtained by using only the deep learning system. In Table 1, we summarize works related to periapical lesion classification. All of them use convolutional neural network.

## 2. Artificial Neural Network

Artificial neural network (ANN) is a field of study of artificial intelligence [40]. The convolutional neural networks are considered a category of artificial neural networks based on deep learning [41]. Their performance approximates the behavior of the receptive fields of the visual cortex [42,43]. CNNs are essentially composed of two characteristic layers: convolutional, which process the inputs as small receptive fields and perform feature extraction; and dense, which are responsible for performing classification according to the features extracted in the convolutional layers [44].

Regarding the task of pattern recognition in medical images, CNNs have been shown to be feasible models with good performance [13]. However, one of the problems usually reported in the use of these networks is the need for high computational capacity and large databases, because of the high number of network parameters [45]. To overcome this challenge, transfer learning is a viable option for dense networks.

### 2.1. Siamese Network

**Figure 1:**
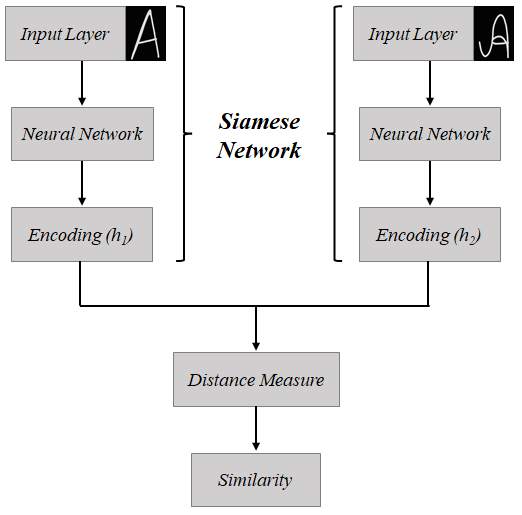
Schematic of the operation of the conventional Siamese Network, used for correspondence between two images. In this case, there is an example, for the correspondence of a letter “A” written in different ways.

The Siamese Network [26], also known as twin network, has the particularity of receiving two images as inputs, which is possible because its architecture has its operation in parallel with two identical subnetworks that are joined at the outputs. The goal of this network is to verify the correspondence between the pair of images; so, the inputs are mapped as feature descriptors that are compared from a similarity function, illustrated in Figure 1, in which $h_{1}$ and $h_{2}$ represent the feature descriptors and the distance measure step is the metric used to verify the similarity.

This class of networks is commonly used to check the similarity between images. It was originally proposed for performing signature verification [46,47,48].

### 2.2. Transfer Learning

Transfer learning aims at the classification process of the current problem by using the “knowledge” learned from a previously network trained in a different dataset (usually larger and more generalist). This “knowledge” is represented by the structure and/or the weights of the convolutional layers. This approach is based on the assumption that previous knowledge acquired in other problems can be useful in solving new ones, as it may be able to find the solution faster and more effectively [49]. Some of the options for pretrained networks are those presented in the computational intelligence competition—the ImageNet Large-Scale Visual Recognition Challenge (ILSVRC) [50]—trained for the publicly available ImageNet dataset, which corresponds to a database of more than fourteen million labeled images, distributed over more than twenty thousand categories; for this reason, it is considered a reference [51].

In the present paper, the VGGNet and DenseNet networks are considered, both trained with the ImageNet dataset and introduced in the ILSVRC in the years of 2014 and 2017, respectively. VGGNet [28] is a CNN architecture with two models, VGG-16 and VGG-19. It was presented by the Visual Geometry Group (VGG) and consists of using filters that are considered small—that is, 3 × 3 in the convolution layers and 2 × 2 in the pooling layers. Transfer learning has been used in many applications. Recently, Barua et al. [52] used pretrained networks for automatic detection of COVID-19: among three different networks used, two are the VGGNet family. Transfer learning in the scenario of a healthcare application is used in the work of Kang, Ullah, and Gwak [53], in which deep learning with transfer learning are used to classify brain tumors in magnetic resonance imaging.

Regarding DenseNet [27], it is a CNN architecture, made available by Facebook AI Research, which can be found in DenseNet-121, DenseNet-169, DenseNet-201, and DenseNet-264. The purpose of this architecture is to promote high connectivity, which was implemented from the connections between layers, so that all subsequent layers can access the outputs of previous layers, i.e., each future layer receives as inputs all the past outputs. The main benefit of using DenseNet is due to resource reuse, which reduces the number of tractable parameters and, as a consequence, the computational complexity [54].

## 3. Imaging Exams in Dentistry

In endodontics, radiographic examination is an indispensable adjunct for diagnosis, treatment, and follow-up after surgical or nonsurgical endodontic therapy. Usually, periapical radiographs are the first choice of imaging method in clinical practice. Nevertheless, some limitations are common and may be a challenge to the diagnosis, such as the compression of three-dimensional anatomy, geometry distortion, and anatomical noise [55,56,57].

The main purpose of nonsurgical and surgical endodontic therapy is to avoid periapical infection or to reverse endodontic periapical or periradicular lesions, thus preventing the spread into the surrounding tissues or healing periapical tissue to maintain the nonvital teeth. The absence or regression of periapical or periradicular radiolucencies and the presence of a sealed root canal are conditions to consider the endodontics therapy successful [57,58].

When tridimensional assessment is required, a Computed Tomography scan is demanded. Commonly, CBCT is a three-dimensional diagnostic image modality used in Dentistry. The development of a relatively small scanner, which allows accurate evaluation of teeth, adjacent tissues, and maxillofacial structures, has made possible to obtain multiplanar reconstructions without magnification using lower radiation doses and having lower cost when compared with Multidetector Computed Tomography (MDCT) [56,59].

During a CBCT acquisition, a cone- or pyramid-shaped X-ray beam and a detector rotate along a circular trajectory. During the rotation, the detector acquires several two-dimensional projections. Then, these projections (raw data) usually undergo preprocessing steps and are reconstructed into a three-dimensional matrix of isotropic voxels of the scanned region, ranging from a small area to the skull. Multiplanar reconstructions (axial, sagittal and coronal views) and other slices are acquired through the reconstructed volume [22].

Generally, CBCT scanners use a low-radiation dose and have higher spatial resolution for hard tissues, especially dental hard tissues and bones. Several CBCT systems are commercially available, with different exposure factors (tube current, exposure time, field of view, kilovoltage) and acquisition parameters (resolution, raw data, and rotation angle), which affect both image quality and radiation dose. The diagnostic task and patient size should be considered in the selection of these acquisition parameters, which can be performed manually or through the selection of preset protocols. Besides, X-ray generation is the other CBCT scanners’ feature that also interferes with the radiation dose. CBCT scanners use continuous or pulsed X-ray generation; the latter allows an exposure time (i.e., the cumulative time during which the patient is exposed to X-ray pulses) considerably smaller than the scan time (i.e., the whole time of acquisition process). Therefore, the reduced motion effect in pulsed scans may result in an improved spatial resolution [22].

Advances in computational techniques and more complex CNNs have contributed to recent advances in Artificial Intelligence (AI). In oral and maxillofacial radiology, CNN models can be used for classification, detection, segmentation, and diagnostic tasks in radiographic image analysis [60,61]. AI may be used as an auxiliary tool in the CBCT scan’s diagnosis for periapical or periradicular radiolucencies detection and for evaluation of endodontic treatment quality.

## 4. Materials and Methods

### 4.1. Database

The study protocol was reviewed and approved by the Local Research Ethics Committee, University of Pernambuco, Brazil (certificate #: 4.881.124).

The initial sample consists of 5343 consecutive CBCT scans from an image database of an Oral Radiology Center of a Dental School in Pernambuco, Brazil. The image database considers all patients referred to the Oral Radiology Center by several professionals for CBCT imaging of the jaws from 2014 to 2017. CBCT scans were acquired using the i-CAT Next Generation (Imaging Sciences International, Inc., Hatfield, PA, USA) operating at 120 kVp, 3-8 mA, field of view (FOV) of 6 × 16 cm, 26 s acquisition time, and 0.13- or 0.40-mm voxel size.

To be included in the study sample, CBCT exams must meet only the following criteria: exams of patients who had at least one endodontically treated maxillary molar. Mandibular CBCT exams and exams with low technical quality and or voxel size greater than 0.20 mm were excluded. After applying the criteria, the final sample was composed of 885 CBCT exams, with a total of 1000 endodontically treated maxillary molars.

**Figure 2:**
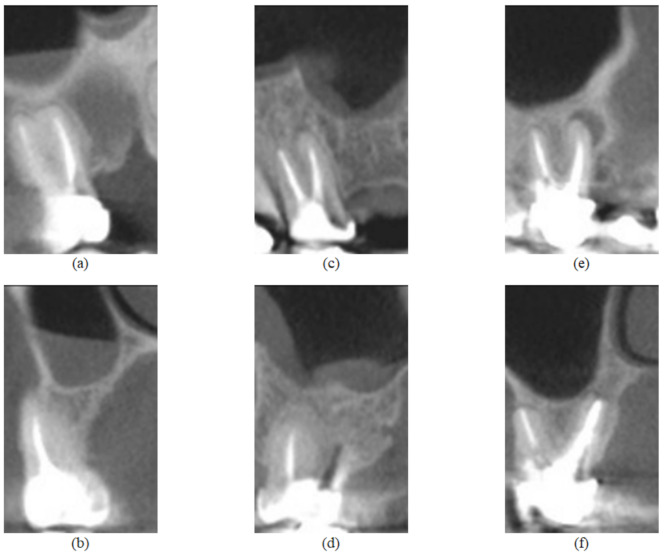
Feature images from the UFPE database, where (**a**,**b**) represent a tooth without lesion and are sagittal and coronal sections, respectively; (**c**,**d**) represent a tooth with small lesion and are sagittal and coronal sections, respectively; (**e**,**f**) represent a tooth with large lesion and are sagittal and coronal sections, respectively.

All CBCT evaluations were performed by an oral and maxillofacial radiologist with 10 years of experience in CBCT diagnosis in a light-dimmed and quiet room using a 24.1 LCD computer monitor (spatial resolution of 1920 × 1200 pixels). The examiner evaluated the entire CBCT volume using the XORAN software (Xoran Technologies, Ann Arbor, MI, USA) and classified the periapical status of each endodontic treated maxillary molar as “presence of periapical lesion” (presence of a well-defined apical radiolucency or 0.5 mm or greater ligament space thickness in more than one multiplanar reconstruction) or “absence of periapical lesion” [58,62]. In addition, when a periapical lesion was present, the examiner measured the extent of the lesion and classified it in one of two groups according to this parameter: small lesions (ranging from 0.5 to 1.9 mm) and big lesions (2.0 mm or greater). These situations are illustrated in Figure 2.

The examiner used TMJ tool to generate sagittal (mesiodistal direction) and coronal (cross-sectional) reconstructions from each tooth. The thickness of the image slices was 1 mm and the distance between slices was 1 mm for both reconstructions (XORAN software, Xoran Technologies, Ann Arbor, MI, USA). The examiner selected the sagittal and coronal reconstructions that demonstrate the periapical status and saved both. A brief summary of the used dataset is presented in Table 2.

### 4.2. Proposed System

The automatic classification system for pairs of sagittal and coronal CBCT slices was implemented from the proposal of a new approach to use for Siamese Network, which we call Siamese Concatenated Network, and transfer learning. The concatenation Siamese Network consists of a network based on the Siamese Network strategy, with the objective of performing a joint analysis of the pair of images. A novelty of the approach is the use of both sagittal and coronal sections, which are jointly evaluated for the presence or absence of periapical lesion. The importance of using a pair of images instead of a single slice comes from the fact that, according to the lesion to be detected, either by location or size, it may be more evident in one of the slices; for this reason, the analysis of both is recommended. Computationally, this situation was corroborated after initial tests, in which only the sagittal or coronal sections were used as input, and the results obtained were below the performance for the concatenated images.

In the Siamese Concatenated Network, pretrained networks based on transfer learning were implemented; this was a decision based on the size of the database, which consists of 1000 evaluations, which can be considered a small number to train the parameters of a deep-learning-based network [45,49]. Thus, the classification system was implemented and evaluated for the DenseNet-121 and VGG-16 networks. The choice of these two networks was due to the fact that they present a superior performance in terms of accuracy compared with the other networks available in the Keras package, which were preliminarily tested. Regarding the database used to perform the training of the parameters, ImageNet was used in both cases.

**Figure 3:**
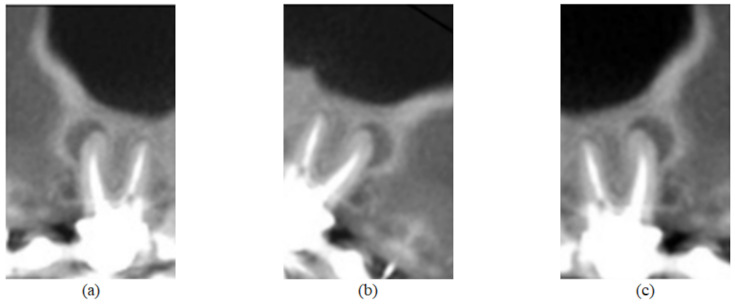
Image of Figure 2e submitted to the three data augmentation techniques used in this work. In (**a**), there is the application of the horizontal flip technique; in (**b**), the rotation technique was applied; and in (**c**), a zoom magnification.

**Figure 4:**
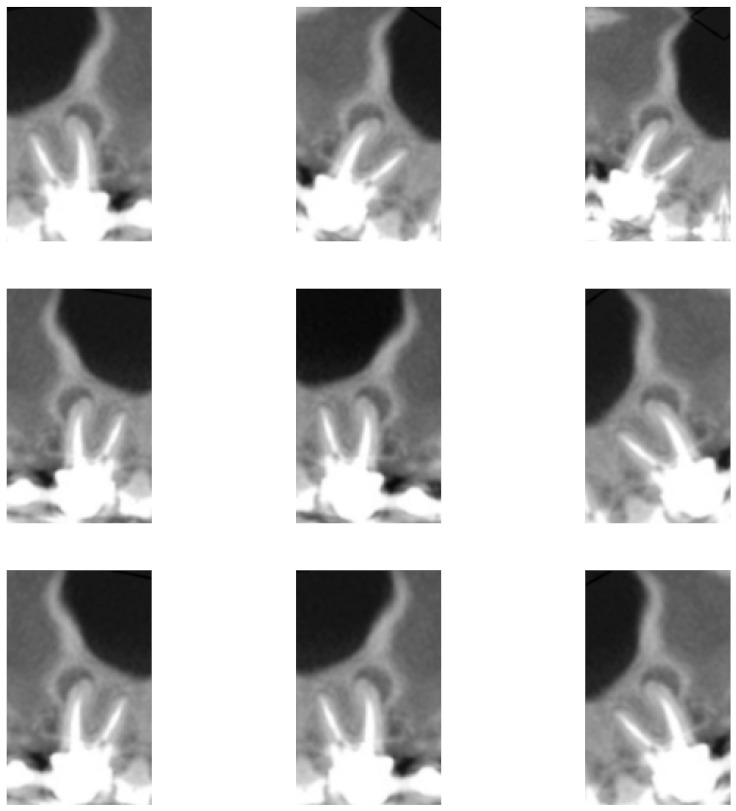
Image of Figure 2e subjected to nine different combinations with the three data augmentation techniques used in this work.

In addition, data augmentation techniques were implemented for the purpose of increasing the number of images to train the network. The adopted techniques and factors were chosen so that the object of analysis would not be compromised—that is, without interfering in the evaluation of the dental roots, preserving the original classification. Thus, three techniques were applied: horizontal flip; rotation, in which a factor of 0.1 was considered—that is, the image was randomly rotated with values in the range $\left[-18^{\circ },18^{\circ }\right]$; and enlargement, also with a factor of 0.1, in which the image is zoomed in and zoomed out, with random values between [−10%,10%] in height, as observed in Figure 3 and Figure 4.

Regarding the images, three different scenarios were evaluated for the proposed system:

- Complete base: 1000 pairs of images are considered. It includes the whole set of images presented in this article—that is, teeth without lesion, teeth with small lesion, and teeth with large lesion.
- Base with large lesions: 724 pairs of images, which correspond to cases of teeth without lesion and teeth with large lesion.
- Base with small lesions: 730 pairs of images, which correspond to cases of teeth without lesions and teeth with small lesion.

For the aforementioned three scenarios, the images were divided into training, validation, and test sets, resulting in 60%, 20%, and 20% of the database, respectively. All tomographic images simulated in this paper have dimensions of 186 × 115 pixels; for each simulation, 150 iterations were fixed, with batch size (amount of training examples considered in an iteration) of 32, with “RMSprop” optimizer and learning rate of 0.001 and the activation function of the output layer was sigmoid.

**Figure 5:**
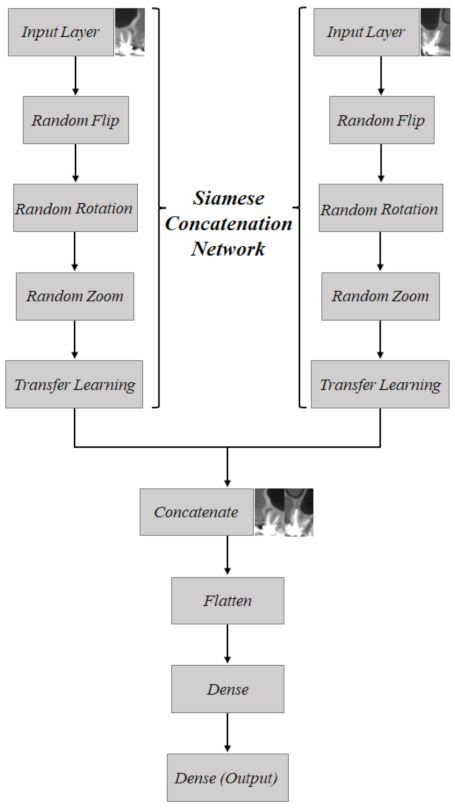
Schematic of how the Siamese Concatenation Network works; a proposed strategy using a Siamese Network for joining pairs of images.

The proposed Siamese Concatenated Network is presented in the methodological scheme presented in Figure 5, in which in the first step the network is duplicated, to process the two distinct inputs, which are the sagittal and coronal tomographic sections. In this first section, the data augmentation techniques were performed (random flip, random rotation, and random zoom, which were previously described) and also the transfer stage learning with the DenseNet-121 and VGG-16 networks. After that, the feature maps generated by the convolutional layers were concatenated to be later classified. Finally, the flatten layer vectorizes the values of the features so that they can be received by the dense layer, which is a fully connected layer with 32 neurons. The dense layer uses the ReLU (Rectified Linear Unit) activation function. The ReLU is a simple nonlinear activation function widely used in deep neural networks. It is given by

(1) $$f\left(x\right)=\left\{\begin{array}{cc} 0, & \mathrm{if}\;x\leq 0\\ x, & \mathrm{otherwise}. \end{array}\right.$$

A dropout of 20% is used after the dense layer that is followed by the output layer, which uses the sigmoid as activation function, given by

(2) $$f\left(x\right)=\frac{1}{1+e^{-x}}.$$

## 5. Results and Discussion

Computational simulations were performed in Python language, considering the strategy of using the Siamese Concatenation Network and the pretrained networks DenseNet-121 and VGG-16. The results were obtained for each of the networks considering the three scenarios of the database use, in other words, full database, large lesion database, as well as small lesion database.

**Figure 6:**
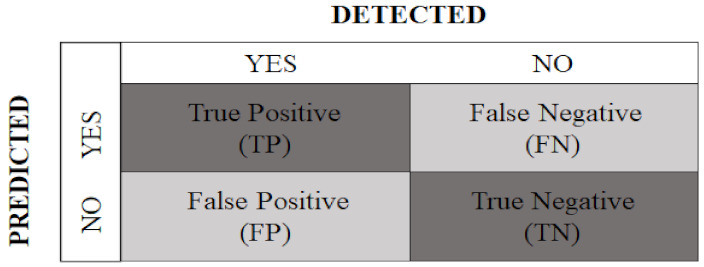
Confusion matrix with predicted classes and detected classes.

To assess the performance of the automated artificial system, metrics related to the confusion matrix were calculated, based on true positives (TP), true negatives (TN), false positives (FP), and false negatives (FN), as shown in Figure 6 [63].

For this, the following metrics were considered.

- Accuracy: provides the percentage of successful classifications, among all those performed, given by
  (3) $$\frac{\mathrm{TP}+\mathrm{TN}}{\mathrm{TP}+\mathrm{TN}+\mathrm{FP}+\mathrm{FN}}.$$
- Recall: reports the percentage of true positive ratings among all true ratings, calculated as follows:
  (4) $$\frac{\mathrm{TP}}{\mathrm{TP}+\mathrm{TN}}.$$
- Precision: indicates the percentage of true positive ratings, among all positive ratings, calculated as follows:
  (5) $$\frac{\mathrm{TP}}{\mathrm{TP}+\mathrm{FP}}.$$
- Specificity: presents the percentage of true negative ratings, among all negative ratings, obtained by
  (6) $$\frac{\mathrm{TN}}{\mathrm{TN}+\mathrm{FN}}.$$
- F1-score: calculated as the harmonic mean between recall and precision:
  (7) $$\frac{2\times \mathrm{recall}\times \mathrm{precision}}{\mathrm{recall}+\mathrm{precision}}.$$

The first evaluation scenario was performed for the UFPE database in its entirety—the 1000 pairs of CT sagittal and coronal sections, which encompasses healthy teeth, as well teeth with large and small periapical lesions.

Table 3 presents the results using all samples in the dataset for the two architectures used in the present paper. Further, it presents the results obtained in [25]. Even though that paper uses a different dataset, we used their reported results for comparison purposes. One may note that the proposed approach using the Siamese Network and transfer learning outperforms the results in [25] for all evaluated metrics. Considering only the proposed approach, it can be inferred that the results obtained for the two networks are similar; however, it is possible to verify that the DenseNet-121 network outperforms the VGG-16 network, since it achieves better accuracy, F1-score, specificity, and precision values, with equal results in recall metrics.

In the second analysis scenario, the dataset consists of all healthy teeth as well those with large lesions—that is, those with lesions larger than or equal to 2 mm in size, which was considered large in this paper. Thus, 724 pairs of tomographic slices were used in this scenario. The results achieved are shown in Table 4.

Based on the analysis of the performance obtained by the networks, it is possible to verify that there is similarity in the results; however, the DenseNet-121 network was superior in three out of the five metrics evaluated, which are F1-score, specificity, and precision, reaching a percentage of 92.39% in specificity. On the other hand, VGG-16 provided the best result in accuracy and recall, reaching 81.25% accuracy.

It is possible to see that the performance considering only teeth without lesions and teeth with lesions considered large is superior compared with the performance for the full base.

The last simulated scenario was for 730 pairs of tomographic slices, which takes into account teeth without lesion and teeth with small lesion, which are in the range of 0.5 to 1.9 mm. The results are shown in Table 5.

According to Table 5, there are similarities in the accuracy performance of the two networks, but with slight superiority of the DenseNet-121 network. In the other metrics evaluated, the DenseNet-121 network was superior in F1-score, specificity, and precision, while it was below the performance of VGG-16 in recall. The results point out the superiority of DenseNet-121, as it performed better in four out of the five metrics measured.

Compared with the performances of the previous scenarios, the performance in this scenario is below the others, which may possibly highlight the fact that very small lesions are difficult to detect, which makes it more complex to distinguish between a tooth with small lesion and a tooth without a lesion.

## 6. Conclusions

In this work, we evaluated techniques for the development of an automatic classification system for endodontic lesions in pairs of cone-beam computed tomography sections, which was implemented using the Siamese Concatenation Network proposed in this paper, based on the Siamese Network, and with the networks DenseNet-121 and VGG-16.

A noteworthy aspect is the pioneering nature of this framework, as no machine-learning-based classification system for dental images with the characteristics considered in this study has been found in the literature. Another aspect to be highlighted is the complexity of the problem, since periapical lesions are not easy to detect. In addition, the lesions present in the images from the UFPE database are considered small for the area of Dentistry (although, in the work, a distinction was made between large and small lesions for the purpose of results analysis), which makes the classification even more complex.

Regarding the classification system, an accuracy of about 70% was obtained for the complete set of images, 81.25% for the set of images without lesions and with large lesions, and 66.67% for the set of images without lesions and with small lesions. The results seem to point to a difficulty that comes from the distinction between teeth without lesions and those with small lesions. To put the results obtained in this work into perspective, in the article by Kruse et al. [64], the performance of human experts in the setting of lesions of already treated teeth in the maxilla showed an accuracy of 63%.

As future works, one may cite the following:

- Introduction of an image segmentation step as part of the classification system. It is worth mentioning that authors [65] report benefits of using image segmentation in machine-learning-based classification systems.
- Application of the proposed Siamese Concatenated Network framework in other classification tasks that involve the use of pairs of images.

## Figures and Tables

**Table 1:** Works related to periapical lesion classification with the use of CNN.

Ref.	Year	CNN Architecture	Dataset	Plane
[23]	2020	U-Net	20 CBCT scan images	Coronal
[24]	2020	U-Net	20 CBCT scan images	Coronal
[25]	2021	U-Net	2902 Panoramic Radiograph images	Coronal
[39]	2021	Not stated	2800 CBCT scan images	Sagittal

**Table 2:** Summary of the used dataset.

Classification	Number of Samples	Features
Healthy	454	Teeth without lesion
Small Lesion	276	Teeth with lesion of 0.5 to 1.9 mm
Large Lesion	270	Teeth with lesion of 2.0 mm or greater

**Table 3:** Results obtained by the compared models considering the test sets for the classification scenario of the entire database. The best values are presented in bold.

Methods			Metrics		
	**Accuracy**	**F1-Score**	**Specificity**	**Precision**	**Recall**
DenseNet-121	**0.7000**	**0.6970**	**0.7634**	**0.7582**	**0.6449**
VGG-16	0.6800	0.6832	0.7204	0.7263	**0.6449**
[25]	—	0.58	—	0.67	0.51

**Table 4:** Performance of the pretrained networks DenseNet-121 and VGG-16 considering the test sets for the classification scenario of 724 images (without lesions and with lesions larger than 2.0 mm) from the UFPE database. The best values are presented in bold.

Methods			Metrics		
	**Accuracy**	**F1-Score**	**Specificity**	**Precision**	**Recall**
DenseNet-121	0.7917	**0.6591**	**0.9239**	**0.8055**	0.5577
VGG-16	**0.8125**	0.6582	0.9100	0.7429	**0.5910**

**Table 5:** Performance of the pretrained networks DenseNet-121 and VGG-16 considering the test sets for the classification scenario of 730 images (without lesions and with lesions between 0.5 and 1.9 mm) from the UFPE database. The best values are presented in bold.

Methods			Metrics		
	**Accuracy**	**F1-score**	**Specificity**	**Precision**	**Recall**
DenseNet-121	**0.6667**	**0.4494**	**0.8571**	**0.6060**	0.3571
VGG-16	0.6599	0.4318	0.8041	0.5000	**0.3800**

## Data Availability

In the present work, a new database was generated, the so-called UFPE Database (Periapical Lesion Classification Dataset), which has 1000 CBCT images. Researchers interested in reproducing the study can request access to the database according to the recommendations at https://github.com/felipebsferreira/periapical-lesion-dataset (accessed on 21 July 2022).

## Abbreviations and Abbreviations

### Abbreviations

UPE	Universidade de Pernambuco
UFRPE	Universidade Federal Rural de Pernambuco
UFPE	Universidade Federal de Pernambuco
CBCT	Cone-Beam Computed Tomography
CNN	Convolutional Neural Network
ANN	Artificial Neural Network
ILSVRC	ImageNet Large-Scale Visual Recognition Challenge
VGG	Visual Geometry Group
AI	Artificial Intelligence
FOV	Field of View
ReLU	Rectified Linear Unit
TP	True Positive
TN	True Negative
FP	False Positive
FN	False Negative
